# The role of nanoliposomal irinotecan plus fluorouracil/leucovorin in the continuum of care of patients with metastatic pancreatic ductal adenocarcinoma

**DOI:** 10.1002/cam4.6111

**Published:** 2023-06-06

**Authors:** Letizia Procaccio, Valeria Merz, Morena Fasano, Vanja Vaccaro, Elisa Giommoni, Andrea Pretta, Silvia Noventa, Maria Antonietta Satolli, Guido Giordano, Clizia Zichi, Carmine Pinto, Camilla Zecchetto, Giulia Barsotti, Ferdinando De Vita, Michele Milella, Lorenzo Antonuzzo, Mario Scartozzi, Alberto Zaniboni, Rosella Spadi, Simona Casalino, Francesca Bergamo, Chiara De Toni, Davide Melisi, Sara Lonardi

**Affiliations:** ^1^ Medical Oncology 1 Unit Veneto Institute of Oncology IOV‐IRCCS Padua Italy; ^2^ Department of Surgery, Oncology and Gastroenterology University of Padua Padua Italy; ^3^ Digestive Molecular Clinical Oncology Research Unit Università degli Studi di Verona Verona Italy; ^4^ Medical Oncology Unit, Department of Precision Medicine, School of Medicine University of study of Campania "L. Vanvitelli" Naples Italy; ^5^ Regina Elena National Cancer Institute Rome Italy; ^6^ Clinical Oncology Unit Careggi University Hospital Florence Italy; ^7^ Medical Oncology Department University Hospital, University of Cagliari Cagliari Italy; ^8^ Medical Oncology Unit Casa di Cura Poliambulanza Brescia Italy; ^9^ Department of Oncology University of Turin, Azienda Ospedaliero‐Universitaria Città della Salute e della Scienza Turin Italy; ^10^ UO di Oncologia Medica Fondazione IRCCS Casa Sollievo della Sofferenza San Giovanni Rotondo Italy; ^11^ Department of Oncology University of Turin, Ordine Mauriziano Hospital Turin Italy; ^12^ High‐Complexity Oncology Unit, Clinical Cancer Center IRCCS Reggio Emilia Reggio Emilia Italy; ^13^ Department of Experimental and Clinical Medicine University of Florence Florence Italy; ^14^ Medical Oncology 1 Azienda Ospedaliero‐Universitaria Città della Salute e della Scienza Turin Italy; ^15^ Investigational Cancer Therapeutics Clinical Unit Azienda Ospedaliera Universitaria Integrata Verona Italy; ^16^ Medical Oncology 3 Unit Veneto Institute of Oncology IOV‐IRCCS Padua Italy; ^17^ Present address: Università degli Studi di Verona Verona Italy

## Abstract

**Background:**

The NAPOLI‐I trial showed better outcome of nanoliposomal irinotecan (nal‐IRI) plus 5‐fluorouracil/leucovorin (5‐FU/LV) compared to 5‐FU/LV in patients with advanced pancreatic ductal adenocarcinoma cancer (advPDAC) progressed to gemcitabine‐based therapy. This study aims to explore the real‐world efficacy and safety of 5‐FU/LV‐nal‐IRI.

**Methods:**

This is a retrospective multicenter analysis including advPDAC patients receiving 5‐FU/LV‐nal‐IRI after failure of gemcitabine‐based therapy. Survival analyses were performed using Kaplan–Meier method, univariate and multivariate analyses by Cox regression.

**Results:**

A total of 296 patients (median age 64.4 years, ECOG PS ≥1 in 56% of cases) were treated at 11 Italian institutions between 2016 and 2018. 34% of them underwent primary tumor resection, and 79% received gemcitabine‐nabpaclitaxel as first line. 5‐FU/LV‐nal‐IRI was administered as second‐line in 73% of cases.

Objective response and disease control rate were 12% and 41%, respectively. Treatment was well tolerated with dose reductions in 50% of patients but no one permanent discontinuation; the commonest grade ≥3 toxicities were neutropenia (14%) and diarrhea (12%). Median PFS and OS from 5‐FU/LV‐nal‐IRI initiation was 3.2 and 7.1 months, respectively.

**Conclusions:**

These real‐world data confirm the 5‐FU/LV‐nal‐IRI efficacy and safety in advPDAC patients progressed to gemcitabine‐based therapy, with outcomes comparable to NAPOLI‐1, even in a less‐selected population and with more modern therapeutic algorithm.

## 1 INTRODUCTION

Pancreatic ductal adenocarcinoma (PDAC) is the third‐leading cause of cancer‐related deaths, and its dismal prognosis is mainly due to late diagnosis, a high recurrence rate after curative resection, and the limited efficacy of approved treatments. Therefore, fewer than 10% of patients survive 5 years after diagnosis.^1^, ^2^, ^3^

The introduction of FOLFIRINOX (oxaliplatin, irinotecan, fluorouracil, and leucovorin) and gemcitabine (Gem) plus albumin‐bound paclitaxel (nab‐paclitaxel, NabP) regimens as front‐line chemotherapy has significantly improved patient outcomes.^4^, ^5^ However, all patients with advanced (adv) PDAC progress under first‐line treatment; therefore, the availability of second‐line options is crucial.^6^, ^7^, ^8^, ^9^ Two randomized phase 3 trials investigated the role of oxaliplatin‐based regimens in this setting, with conflicting results.^10^, ^11^

In 2015, nanoliposomal irinotecan (nal‐IRI, an intravenous liposomal formulation of the topoisomerase I inhibitor irinotecan) in combination with 5‐fluorouracil (5‐FU) and folinic acid (leucovorin, LV) was approved by the Food and Drug Administration (FDA) in patients previously treated with Gem‐based chemotherapy on the basis of the results of the NAPOLI‐1 phase III trial.^12^ In this study, 417 patients with advPDAC were randomized to three treatment arms and the treatment with nal‐IRI and 5‐FU/LV demonstrated superior survival and maintenance of quality of life compared to 5‐FU/LV monotherapy. These benefits were also maintained over an extended follow‐up period.^13^, ^14^

These new effective first‐ and second‐line treatment options in advPDAC sparked a debate over the optimal continuum of care for the management of this hard‐to‐treat disease.

Data collected outside a controlled trial provide useful additional information regarding the clinical use, efficacy, and safety of drugs when used in a real‐world setting; however, reports about post‐approval use of nal‐IRI plus 5‐FU/LV are still uncommon and have a small sample size.^15^, ^16^, ^17^, ^18^, ^19^, ^20^, ^21^ Therefore, there is still a critical need to learn more about this regimen's performance and feasibility in daily practice and its optimal use in the therapeutic algorithm.

In many countries, such as in Italy, nal‐IRI is not yet an available option despite the approval by American and European regulatory agencies. Following the FDA's approval of 5‐FU/LV‐nal‐IRI in 2015, pretreated patients with advPDAC in Europe were granted early access through a compassionate use program (CUP). Herein, we present the results from a large Italian multicenter study on the use of 5‐FU/LV‐nal‐IRI in advPDAC patients.

## 2 METHODS

This was a multicenter, observational, retrospective study including patients with histologically proven advPDAC who received 5‐FU/LV‐nal‐IRI after failure of a Gem‐based therapy within a nominal use program from 2016 to 2018. All patients consecutively treated in the 11 participating Italian centers were enrolled to avoid selection bias. The Coordinating Site's institutional board and all the Ethics Committees involved gave their approval for the study. All participants gave their written informed consent in accordance with the Declaration of Helsinki.

The use of a shared database was approved by all the authors and clinical, biomolecular, and pathological variables were carefully defined to avoid bias in data reporting. Data were collected by local investigators but centrally reviewed. Demographics and patient characteristics were summarized through descriptive statistics.

To assess 5‐FU/LV‐nal‐IRI activity, the individual patient response was evaluated every 8–12 weeks by CT scan as per clinical practice, using RECIST version 1.1 criteria. The objective response rate (ORR) represented the percentage of patients with a complete response (CR) or partial response (PR), while the disease control rate (DCR) represented the percentage of patients with a CR, PR, or stable disease (SD).

Progression‐free survival (PFS) was measured from the 5‐FU/LV‐nal‐IRI start to progression, relapse, or death from any cause and was censored at the date of the last available follow‐up. Overall survival (OS) was defined as the time from the first 5‐FU/LV‐nal‐IRI dose to death from any cause and was censored at the date of the last available follow‐up.

PFS2 and OS2 in patients who received gemcitabine‐nabpaclitaxel (Gem‐NabP) as first‐line followed by nal‐IRI plus 5‐FU/LV as second‐line were evaluated to assess the efficacy of the entire first‐ and second‐line strategies. PFS2 was defined as the time from the first Gem‐NabP dose to the date of disease progression on nal‐IRI plus 5‐FU/LV administered after the first disease progression or death. OS2 was defined as the time from the initiation of Gem‐NabP to the date of death from any cause. Survival functions were estimated using the Kaplan–Meier method and compared using the log‐rank test. Median follow‐up was calculated by Kaplan–Meier inverse method. The Cox proportional hazards model was used for both univariate and multivariate analyses of OS and PFS. Only the variables that were statistically significant in the univariate analysis were imputed in the multivariate analysis.

Explorative analysis was performed to identify potential prognostic factors.

Safety and tolerability were evaluated by classifying adverse events (AEs) according to the NCI CTCAE version 4.0.

No formal sample size estimation and power calculation were made for this retrospective study.

Statistical analyses were performed using the open‐source statistical software package R 4.2.0, and a two‐sided *p* value ≤0.05 was considered statistically significant.

## 3 RESULTS

A total of 296 patients were treated, with 149 males (50.3%) and a median age of 64.4 years (range 30.1–82.7). Eastern Cooperative Oncology Group performance status (ECOG PS) was ≥1 in 55.7% of cases; body mass index (BMI) was ≤18.5 in 17.9% of subjects.

Primary tumors were previously resected in 100 out of 296 patients (33.8%), and neo‐adjuvant, adjuvant therapy, or both were administered in 26% of cases. Table 1 summarizes the patient and tumor characteristics.

**TABLE 1:** Patients and tumor characteristics at 5‐FU/LV‐nal‐IRI initiation, and treatments received before 5‐FU/LV‐nal‐IRI administration.

Characteristic	Total = 296
	*N* (%)
Age at start of treatment	
Median (range)	64.4 (30.1–82.7)
≥70	91 (30.7%)
Baseline ECOG PS	
0	131 (44.3%)
1	141 (47.6%)
2	24 (8.1%)
BMI	
≤18.5	53 (17.9%)
Albumin	
<UNL (4 g/dL)	73 (24.6%)
Total bilirubin	
>UNL (17 umol/L)	3 (1.0%)
Baseline CA 19.9	
>UNL (37 ng/mL)	227 (76.7%)
Primary tumor location	
Head/Uncinated process	172 (58.1%)
Other	124 (41.9%)
Stage at PDAC diagnosis	
I–II	76 (25.7%)
III	69 (23.3%)
IV	151 (51.0%)
Primary tumor resected	100 (33.8%)
Biliary stenting any time	81 (27.4%)
Number of site of disease	
1	124 (41.9%)
>1	172 (58.1%)
Sites of disease	
Liver	211 (71.3%)
Distant lymph node	115 (38.9%)
Peritoneum	76 (25.7%)
Lung	101 (34.1%)
Pancreas	64 (21.6%)
Bone	13 (4.4%)
Other	15 (5.1%)
Therapy for non‐metastatic disease	
Adjuvant	55 (18.6%)
Neo‐adjuvant	22 (7.4%)
Radiotherapy on primary tumor	42 (14.2%)
Previous lines for metastatic disease	289 (97.6%)
Gemcitabine monotherapy	8 (2.7%)
Gemcitabine plus nab‐paclitaxel	234 (79.1%)
FOLFIRINOX	26 (8.8%)

At basal evaluation for treatment with nal‐IRI plus 5‐FU/LV, 11 subjects (3.7%) had locally advanced PDAC with unresectable disease (6) or local relapse after previous resection (5), while 43 patients (14.5%) presented both local disease and distant metastases. Globally, 58.1% of cases had more than one metastatic site of disease.

Gem‐NabP was administered as first‐line treatment in 234 (79.1%) patients. The 5‐FU/LV‐nal‐IRI regimen was administered as second‐line therapy in 72.3% of patients, while in 23% and 2.4% of cases it was administered as third‐ and fourth‐line, respectively. A total of 198 subjects (66.9%) received the sequence Gem‐NabP as a first‐line treatment and 5‐FU/LV‐nal‐IRI as second‐line (Table 1).

With regard to 5‐FU/LV‐nal‐IRI activity, 36 patients achieved an objective response (12.2% ORR) and 4 of them (1.3%) a complete response. A total of 84 subjects (28.4%) achieved stable disease, with a global DCR of 41.1% lasting a median duration of 6.2 months (range 0.7–61.5). At the 2‐ to 3‐month evaluation, the ECOG PS improved (10.1%) or was maintained (41.9%) in a total of 154 patients (52%) (Table 2).

**TABLE 2:** Radiological and clinical response to 5‐FU/LV‐nal‐IRI.

Characteristic	Total = 296
	*N* (%)
Objective response rate	
Complete response	4 (1.3%)
Partial response	32 (10.9%)
Stable disease	84 (28.4%)
Progression disease	172 (58.1%)
MD	4 (1.3%)
Disease control rate	120 (41.1%)
Clinical benefit	
ECOG PS improvement	30 (10.1%)
Maintenance ECOG PS0	124 (41.9%)

**FIGURE 1:**
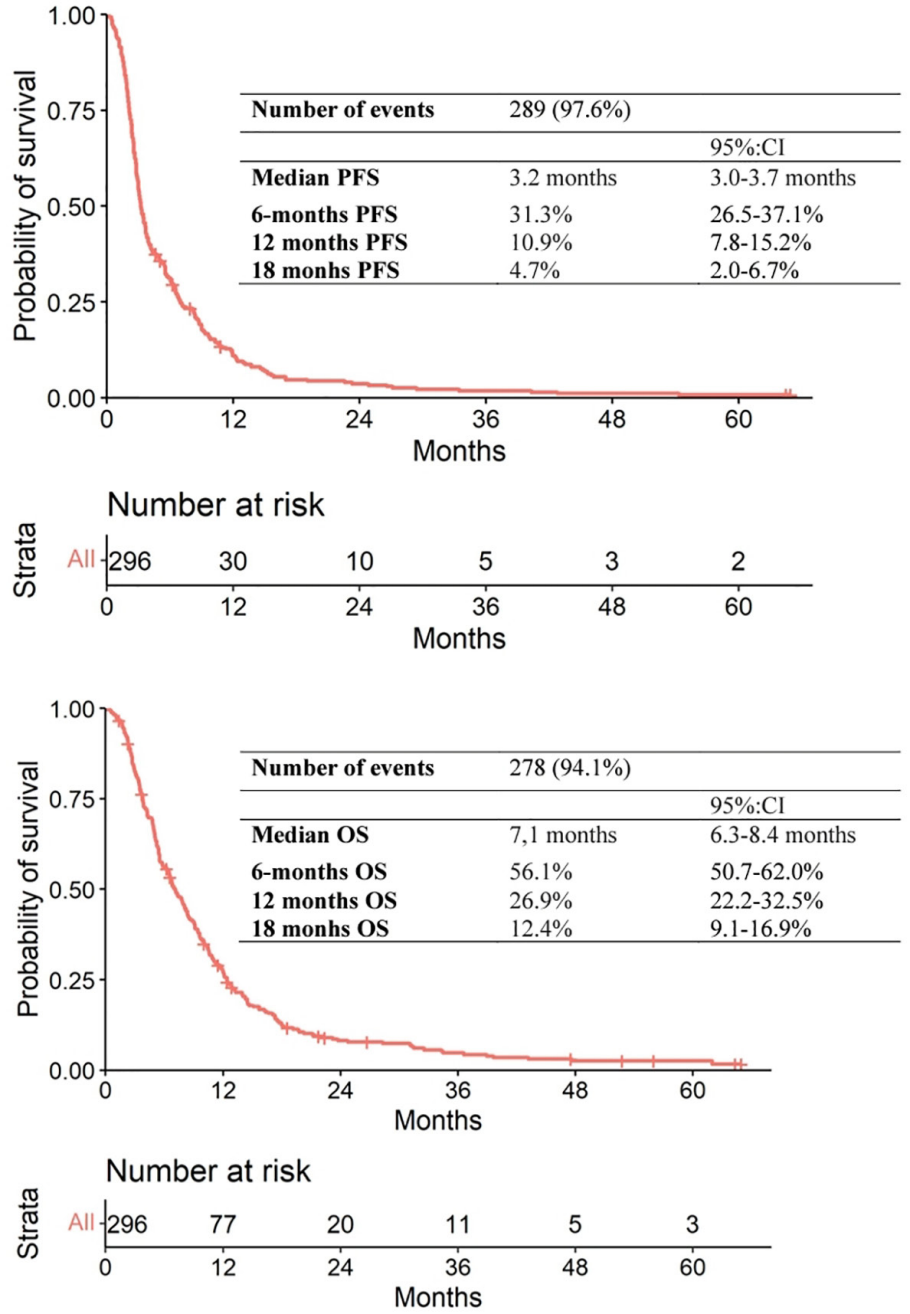
Kaplan–Meier curve for progression‐free survival and overall survival (*n* = 296). PFS, progression‐free survival; OS, overall survival.

At the time of the present analysis, 289 patients out of 296 had progressed to 5‐FU/LV‐nal‐IRI and the median (m) PFS was 3.2 months (95% CI: 3–3.7). PFS estimates were 31.3% and 10.9% at 6 and 12 months, respectively (Figure 1). In 66.9% of the study population receiving the 5‐FU/LV‐nal‐IRI regimen as a second‐line treatment after Gem‐NabP had failed, the mPFS2 was 12.4 months (95% CI: 11.6–13.3).

Out of 296 patients, 278 died with a mOS of 7.1 months (95% CI: 6.3–8.4) from the start of 5‐FU/LV‐nal‐IRI, with a median follow‐up of 56 months. OS probabilities stood at 56.1% at 6 months, 26.9% at 12 months, and 12.4% at 18 months (Figure 1). Three known unfavorable prognostic factors were confirmed at a multivariate analysis: poor ECOG PS (HR: 1.49; 95% CI: 1.2–1.9; *p* = 0.003), high CA: 19.9 (HR: 1.63; 95% CI: 1.2–2.3; *p* = 0.005), and omission of neo‐adjuvant therapy (HR: 2.25; 95% CI: 1.2–4.3; *p* = 0.01) (Table 3; Table S1).

**TABLE 3:** Multivariable Cox regression model for OS from 5‐FU/LV‐nal‐IRI treatment start.

Characteristic		OS		
		HR	95% CI	*p*‐Value
Primary tumor resected (ref: Yes)	No	1.09	0.73–1.63	0.6740
Time to metastases (ref: Metachronous)	Synchronous	1.07	0.71–1.60	0.7500
Primary tumor location (ref: Other)	Head /Uncinated process	0.86	0.66–1.12	0.2552
Number of metastatic sites (ref: 1)	>1	1.21	0.93–1.57	0.1510
Previous anticancer therapy for non‐metastatic disease: adjuvant (ref: Yes)	No	1.62	0.95–2.74	0.0754
Previous anticancer therapy for non‐metastatic disease: neo‐adjuvant (ref: Yes)	No	2.25	1.18–4.27	**0.0135**
Baseline ECOG PS (ref: 0)	≥1	1.49	1.15–1.92	**0.0026**
Baseline CA 19.9 (ref: ≤37 mg/mL)	>UNL (37 ng/mL)	1.63	1.16–2.30	**0.0052**

*Note*: The model was stratified for the variable “Neutrophil‐to‐lymphocyte ratio (NLR),” as it does not respect the assumption of proportionality of the risks.

**FIGURE 2:**
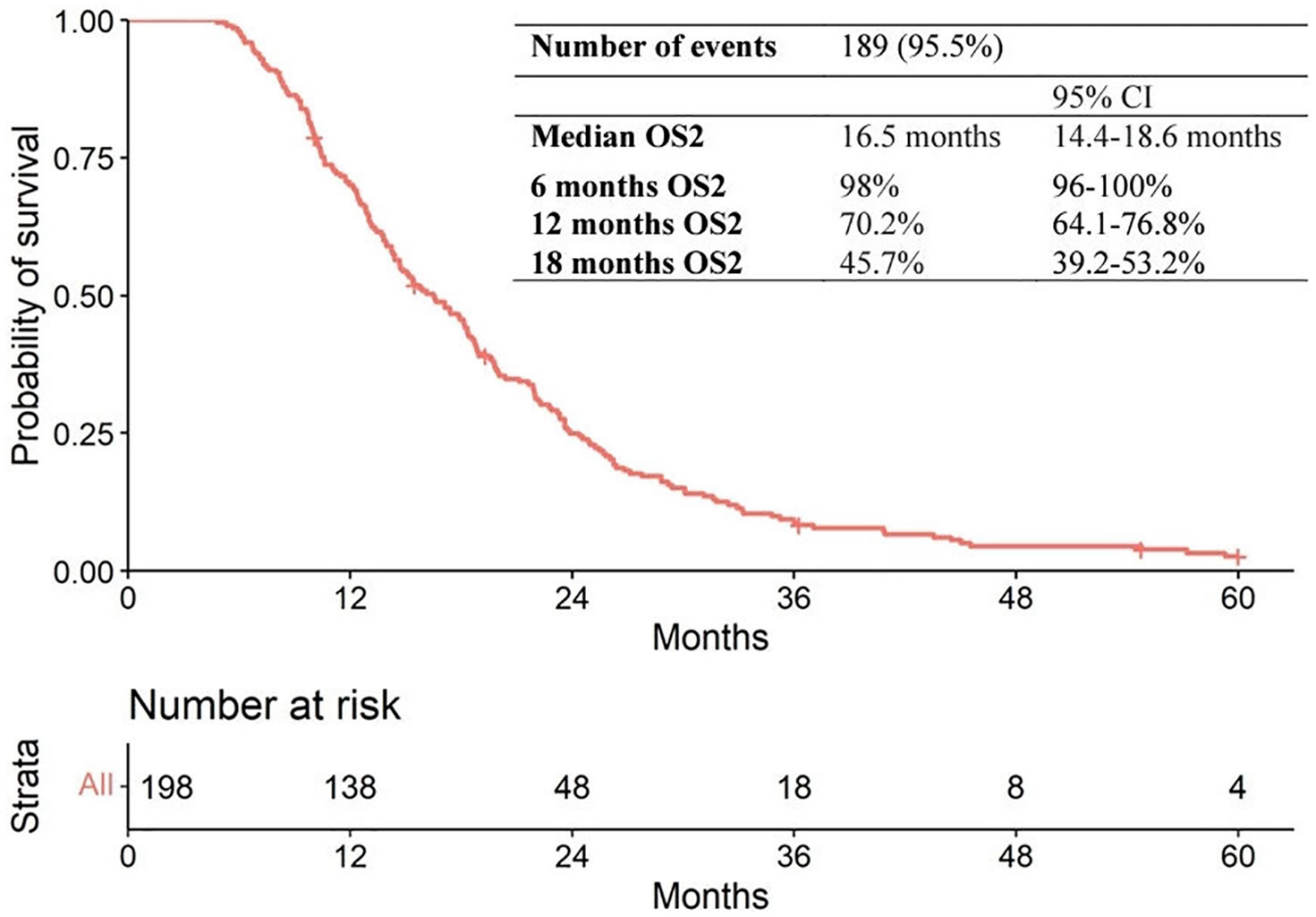
Kaplan–Meier curve for overall survival from the start of first line to death in patients receiving the sequence of treatments Gem‐NabP ➔ 5‐FU/LV‐nal‐IRI (*n* = 198**).** OS, overall survival; Gem‐NabP, gemcitabine‐nabpaclitaxel.

Globally, the mOS from PDAC diagnosis was 21.8 months (95% CI: 20–25.4) with a median follow‐up of 86.3 months. The mOS from first diagnosis of advanced disease was 17.9 months (95% CI: 16.2–19.9) with a median follow‐up of 65.1 months; probability of survival was 75.6% and 33.8% at 1 and 2 years, respectively. In 66.9% of the study population receiving 5‐FU/LV‐nal‐IRI as a second‐line therapy after Gem‐NabP had failed, the mOS2 was 16.5 months (95% CI: 14.4–18.6) (Figure 2) with a median follow‐up of 60 months.

The median number of administered cycles was 5 (IQR: 3–9), with 213 patients (72.4%) receiving at least 4 cycles and 120 subjects (40.8%) receiving at least 6 cycles. The median and mean time on 5‐FU/LV‐nal‐IRI treatment were 2.5 (IQR: 1.5–5.7) and 4.5 (range: 1–61.5) months.

A total of 148 patients (50%) experienced at least one dose reduction, and the administration of 5‐FU/LV‐nal‐IRI was delayed at least once in 36.6% of cases.

The safety profile was as expected, with 87 patients (29.4%) having a grade ≥3 adverse event. The most frequent toxicities with a ≥3 grade were the following: neutropenia (13.5%), with 1 febrile case and 50 cases (16.8%) undergoing granulocyte colony stimulating factor administration, diarrhea (11.5%), fatigue (3%), and anemia (3%). No toxic deaths were reported, but 32 patients (10.8%) died within 30 days of the last 5‐FU/LV‐nal‐IRI cycle due to disease progression (Table 4).

**TABLE 4:** 5‐FU/LV‐nal‐IRI treatment disposition and toxicity.

Characteristic	Total = 296
	*N* (%)
Setting of nal‐IRI‐5FULV administration	
First‐line	7 (2.4%)
Second‐line	214 (72.3%)
Third‐line	68 (23%)
Fourth‐line	7 (2.4%)
Number of cycles	
Median (IQR)	5 (3–9)
≥4 cycles	213 (72.4%)
≥6 cycles	120 (40.8%)
Time on treatment (months)	
Median (IQR)	2.5 (1.5–5.7)
Mean (range)	4.5 (1–61.5)
<6 months	228 (77.6%)
≥6 months	66 (22.4%)
≥12 months	20 (6.8%)
≥18 months	7 (2.4%)
Patients experiencing at least one dose reduction	148 (50.0%)
Patients experiencing at least one delay	107 (36.6%)
Patients experiencing at least one adverse Events ≥G3	87 (29.4%)
Neutropenia ≥G3	40 (13.5%)
Febrile neutropenia	1 (0.3%)
Anemia ≥G3	9 (3%)
Diarrhea ≥G3	34 (11.5%)
Fatigue ≥G3	9 (3%)
Nausea	6 (2%)
Vomiting	4 (1.4%)

## 4 DISCUSSION

The usefulness and the choice of the optimal regimen in the second‐line treatment for advPDAC following Gem‐based therapy are still under debate, and the decision in clinical practice is largely made on a case‐by‐case basis.^8^, ^9^

The NAPOLI‐1 trial established a new standard therapy in pretreated advPDAC,^12^, ^14^ and nal‐IRI is currently under clinical investigation with oxaliplatin and 5‐FU/LV in a first‐line randomized phase 3 trial in advPDAC and in a phase 2 study of perioperative treatment in resectable PDAC.^22^ Despite that, the use of nal‐IRI in European daily practice is still limited, mainly due to registration constraints. To our knowledge, the present study is the largest and most detailed report on the efficacy and safety of nal‐IRI in a real‐world population.

Our observations from real‐life clinical practice revealed activity consistent with the findings of the NAPOLI‐1 study: the ORR was 12.2% and the DCR 41.1% versus the trial's 17% and 49%, respectively.^12^ The outcomes of our analysis were also comparable to the results reported in the randomized study, with a mPFS of 3.2 versus 3.1 months. Regardless of the comparable PFS, data should be interpreted with caution since the time to first tumor assessment in real life is longer than in the prospective trial. Also, the mOS was consistent with that of NAPOLI‐1 at 7.1 versus 6.1 months. The efficacy of 5‐FU/LV‐nal‐IRI was thus reproducible in 11 different centers across Italy and in a less favorably selected population: our cohort was older (age range: 30–83 vs. 57–70 years) and had worse general conditions (ECOG PS ≥1: 56% vs. 41%) than the pivotal trial.^12^, ^13^ Six‐ and 12‐month PFS and OS rates were in line with both the NAPOLI‐1 trial^12^, ^13^ and real‐world analyses.^15^, ^16^, ^17^, ^18^, ^19^, ^20^, ^21^ With respect to prior treatment, only 55% of patients in the NAPOLI‐1 trial received Gem combination treatment,^12^, ^13^ but most patients (79%) in our study were treated with Gem‐NabP as first‐line. Similarly, compared to several other retrospective analyses,^15^, ^16^, ^17^, ^18^, ^19^, ^20^, ^21^ our cohort was more homogeneous in terms of previous treatments. Compared to the randomized trial and real‐world evidence, our findings support the role of 5‐FU/LV‐nal‐IRI even in a more modern therapeutic algorithm.

Recent real‐world experiences include the analysis by Park et al.,^20^ which was the first to evaluate survival outcomes from first‐line initiation in 94% of advPDAC patients who first received Gem‐NabP and subsequently 5‐FU/LV‐nal‐IRI as second‐line at first disease progression. The authors reported a PFS2 and OS2 similar to those calculated in our cohort study (PF2: 13.8 vs. 12.4 months; OS2: 16.3 vs. 16.5 months), but with a very small sample size including only 51 patients.

Compared to prospective trials investigating options as a second‐line regimen^10^, ^11^, ^23^, ^24^ or to propensity score analysis,^25^ sequential treatment of nal‐IRI after Gem‐NabP appears to be a reasonable strategy with similar survival outcomes from first‐line initiation but with more manageable toxicities not overlapping with possible residual neurotoxicity from Gem‐NabP regimen previously received. A comparative randomized trial is needed to confirm the optimal sequential strategy for advPDAC patients.

Our study revealed a relevant proportion of patients who sustained long‐lasting benefits (12‐month OS from the start of 5‐FU/LV‐nal‐IRI: 27%; 18‐month OS: 12%), suggesting the existence of a subgroup with favorable prognostic factors or biomarkers who thus gain a higher benefit from therapy. The identification of such clinical or molecular characteristics may be helpful in selecting patients for the treatment.

As expected, in our analysis patients having a good PS and/or a low CA 19.9 demonstrated a better prognosis. Previous neo‐adjuvant therapy administration (*N* = 22, 7.4%) also confirmed its positive impact, independently from other favorable clinical factors included in the model (primary tumor resection, metachronous metastases), probably as combined expression of both patient and tumor good prognosis features. In a recent study, IL‐8 emerged as a potential predictive biomarker of resistance to nal‐IRI.^26^

Another interesting finding emerged from the analysis of global survival from the initial diagnosis of PDAC and advPDAC, which was 22 and 18 months, respectively: these values are better than expected and may reinforce the clinical relevance of 5‐FU/LV‐nal‐IRI in the sequential treatment of advPDAC, even in the absence of sufficient data for a proper comparison with other combination regimens.^10^, ^11^

No new safety concerns were detected with 5‐FU/LV‐nal‐IRI in this real‐world study. As in the NAPOLI‐1 trial,^12^, ^13^ the most common grade 3 toxicities were neutropenia, diarrhea, fatigue, and anemia. Although G‐CSF prophylaxis was not part of the pivotal trial, 37% of the NAPOLI‐1 population experienced neutropenia.^12^, ^13^ Interestingly, 17% of the patients in our cohort received G‐CSF in accordance with the guidelines for its use in regimens with more than a 20% risk of neutropenia.^27^ With respect to dose modifications, the dose of 5‐FU/LV‐nal‐IRI was reduced in 50% of our patient cohort with no significant impact on clinical outcomes.^28^ This is consistent with the updated results from the NAPOLI‐1 study^12^, ^13^ and those reported by Glassman et al.^17^ and Park et al.,^20^ demonstrating that dose reductions did not significantly affect survival. This suggests that appropriate dose modifications of nal‐IRI plus 5‐FU/LV should be considered in clinical practice for patients on longer treatment regimens.

Our study has some limitations, first due to its observational and retrospective design.^29^ As is common knowledge, some toxicity data and outcomes may be less rigorously reported in clinical charts, which may cause for example an underestimation of AEs in our report. Second, the choice of nal‐IRI setting was at the clinician's discretion, leading to a relative heterogeneity of the study population in terms of line of nal‐IRI administration. On the contrary, this cohort was the most homogeneous to date with respect to prior therapies due to 68% of patients receiving an up‐to‐date sequence of Gem‐NabP as first‐line and 5‐FU/LV‐nal‐IRI as second‐line treatments.

## 5 CONCLUSION

As nal‐IRI is now under evaluation by Regulatory Agencies in some European countries, real‐world data are of huge importance to estimate its real benefit in clinical practice.

Our large multicenter data confirmed the efficacy and safety of 5‐FU/LV‐nal‐IRI in patients with advPDAC progressed to a Gem‐based therapy, with outcome comparable to NAPOLI‐1 even in a less selected population treated with a more active first‐line therapy. Nal‐IRI plus 5‐FU/LV had a favorable safety profile, not overlapping with possible residual neurotoxicity from Gem‐NabP previously received.

In this new continuum of care, 5‐FU/LV‐nal‐IRI could stand as a valuable addition to the scarce arsenal of treatments for advPDAC. The presence of a subgroup of patients with long survival (27%) underlines that even in advPDAC there is now room of significant survival prolongation, and this new second‐line therapy should be offered to the largest number of subjects. Nonetheless, the selection of the best first‐line, the identification of the right window to start a second line, the attention to clinical prognostic factors, and the early simultaneous care approach are in our opinion the pillars for maximizing the benefit of 5‐FU/LV‐nal‐IRI for every single patient.

## AUTHOR CONTRIBUTIONS

**Letizia Procaccio:** Conceptualization (equal); data curation (equal); formal analysis (supporting); funding acquisition (lead); investigation (equal); methodology (equal); project administration (equal); resources (equal); software (supporting); supervision (equal); validation (equal); visualization (equal); writing – original draft (equal); writing – review and editing (equal). **Valeria Merz:** Conceptualization (equal); data curation (equal); investigation (equal); methodology (equal); project administration (equal); resources (equal); validation (equal); visualization (equal); writing – original draft (equal); writing – review and editing (equal). **Morena Fasano:** Data curation (equal); investigation (equal); resources (equal); visualization (equal). **Vanja Vaccaro:** Data curation (equal); investigation (equal); resources (equal); visualization (equal). **Elisa Giommoni:** Data curation (equal); investigation (equal); resources (equal); visualization (equal). **Andrea Pretta:** Data curation (equal); investigation (equal); resources (equal); visualization (equal). **Silvia Noventa:** Data curation (equal); investigation (equal); resources (equal); visualization (equal). **Maria Antonietta Satolli:** Data curation (equal); investigation (equal); resources (equal); visualization (equal). **Guido Giordano:** Data curation (equal); investigation (equal); resources (equal); visualization (equal). **Clizia Zichi:** Data curation (equal); investigation (equal); resources (equal); visualization (equal). **Carmine Pinto:** Data curation (equal); investigation (equal); resources (equal); visualization (equal). **Camilla Zecchetto:** Data curation (equal); investigation (equal); resources (equal); visualization (equal). **Giulia Barsotti:** Data curation (equal); investigation (equal); resources (equal); visualization (equal). **Ferdinando De Vita:** Data curation (equal); investigation (equal); resources (equal); visualization (equal). **Michele Milella:** Data curation (equal); investigation (equal); resources (equal); visualization (equal). **Lorenzo Antonuzzo:** Conceptualization (equal); investigation (equal); resources (equal); visualization (equal). **Mario Scartozzi:** Data curation (equal); investigation (equal); resources (equal); visualization (equal). **Alberto Zaniboni:** Data curation (equal); investigation (equal); resources (equal); visualization (equal). **Rosella Spadi:** Data curation (equal); investigation (equal); resources (equal); visualization (equal). **Simona Casalino:** Data curation (equal); investigation (equal); resources (equal); visualization (equal). **Francesca Bergamo:** Conceptualization (equal); data curation (equal); investigation (equal); methodology (equal); project administration (equal); resources (equal); supervision (equal); visualization (equal); writing – review and editing (equal). **Chiara De Toni:** Data curation (equal); formal analysis (lead); investigation (equal); methodology (equal); software (equal); validation (equal); visualization (equal). **Davide Melisi:** Conceptualization (equal); data curation (equal); investigation (equal); methodology (equal); project administration (equal); resources (equal); supervision (equal); validation (equal); visualization (equal); writing – original draft (equal); writing – review and editing (equal). **Sara Lonardi:** Conceptualization (equal); data curation (equal); formal analysis (supporting); investigation (equal); methodology (equal); project administration (equal); resources (equal); software (supporting); supervision (lead); validation (equal); visualization (equal); writing – original draft (equal); writing – review and editing (lead).

## FUNDING INFORMATION

This study was partially funded by “Progetto di Biopsia Liquida P31” CDC 099139 (5X1000‐2018: Cancer Genomics Research Platform).

## CONFLICT OF INTEREST STATEMENT

The authors declare that they have no known competing financial interests or personal relationships that could have appeared to influence the work reported in this paper. The authors declare the following financial interests/personal relationships which may be considered as potential competing interests: LP: personal fee for scientific consultancy from Astra Zeneca. EG: personal fee for scientific consultancy from Amgen, Astra Zeneca, Viatris, Servier; invited speaker from Amgen, Viatris, Servier. MAS: personal fee from Astra Zeneca, Servier, BMS. GG: personal fee for scientific consultancy from Astra Zeneca, MSD; invited speaker from Servier, Ipsen, Novartis, Bayer, Astra Zeneca, MSD, BMS, Seagen, Amgen. CP: personal fee for scientific consultancy and invited speakers from Amgen, Astellas, Astra Zeneca, Bayer, BMS, Celgene, Clovis Oncology, Eisai, Ipsen, Janssen, Incyte, Merck Serono, MSD, Novartis, Roche, Sandoz, Sanofi, Servier. FDV: personal fee for scientific consultancy for Servier, Lilly, MSD and BMS; invited speaker from Roche, Bayer, Servier, Lilly, Astellas, MSD and BMS. MM: personal fee for scientific consultancy from Viatris, Astrazaneca, MSD, Merck Serono; Data Monitoring Committees: Novartis. LA: personal fee as invited speaker from Roche, Astra Zeneca, Novartis, IPSEN, Amgen, Lilly, Pfizer, Merk, MSD. MS: personal fees from MSD, Merck, Eisai, Sanofi, Bayer, Servier. AZ: personal fee as invited speaker from Amgen, MSD, Pierre Fabre. FB: personal fee for scientific consultancy from Servier, AAA Novartis; invited speaker from Lilly, MSD, EISAI, Bayer. DM: personal fees for scientific consultancy from Shire, Incyte, Servier, iOnctura, Baxter, Eli Lilly, Evotec. SL: personal fee for scientific consultancy from Amgen, Astra Zeneca, BMS, Daiichi‐Sankyo, Incyte, Lilly, Merck Serono, MSD, Servier; invited speaker from Amgen, BMS, Incyte, GSK, Lilly, Merck Serono, MSD, Pierre‐Fabre, Roche, Servier. VM, MF, VV, AP, SN, CZ, CZ, GB, RS, SC, CDT: no interest to declare.

## ETHICAL APPROVAL

All participants gave their written informed consent in accordance with the Declaration of Helsinki.

## CONSENT TO PARTICIPATE

The Coordinating Site's institutional board (CESC IOV 26/02/2018) and all the Ethics Committees involved gave their approval for the study.

## Supporting information


Table S1.
Table S2.
Table S3.
Table S4.
Table S5.

## Data Availability

All analyzed data could be available on request.
