# Supplementary material for: The role of nanoliposomal irinotecan plus fluorouracil/leucovorin in the continuum of care of patients with metastatic pancreatic ductal adenocarcinoma
Source: Cancer Med. 2023 Jun 6;12(13):14337–45. doi: 10.1002/cam4.6111 (PMC10358242; doi:10.1002/cam4.6111)
Supplement: Supplementary file 1 — Table S1. Table S2. Table S3. Table S4. Table S5. [file CAM4-12-14337-s001.docx]

**SUPPLEMENTARY MATERIAL**

**Table 1_S. Univariate analyses for OS of baseline characteristics**

| **Characteristic** | | **Overall Survival** | | |
| --- | --- | --- | --- | --- |
|  |  | **HR** | **95% CI** | **p** |
| Sex | Female | Ref. |  |  |
|  | Male | 1.06 | 0.84 – 1.34 | 0.6251 |
| Age (70 years cut-off) | < 70 | Ref. |  |  |
|  | ≥ 70 | 0.94 | 0.73 – 1.21 | 0.6168 |

**Table 2_S. Univariate analyses for OS of tumor characteristics**

| **Characteristic** | | **Overall Survival** | | |
| --- | --- | --- | --- | --- |
|  |  | **HR** | **95% CI** | **p** |
| Primary tumor resected | No | Ref. |  |  |
|  | Yes | 1.83 | 1.41 – 2.37 | **<0.0001** |
| Primary tumour location | Head /Uncinated process | Ref. |  |  |
|  | Other | 0.79 | 0.62 – 1.00 | **0.0495** |
| T | 1-2 | Ref. |  |  |
|  | 3-4 | 1.14 | 0.80 – 1.62 | 0.4808 |
| N | 0 | Ref. |  |  |
|  | >0 | 0.95 | 0.67 – 1.34 | 0.7691 |
| Grading | G1-G2 | Ref. |  |  |
|  | G3-G4 | 0.83 | 0.64 – 1.07 | 0.1484 |
| Histological type | Ductal adenocarcinoma | Ref. |  |  |
|  | Other | 1.76 | 0.87 – 3.57 | 0.1155 |
| Time to metastases | Synchronous | Ref. |  |  |
|  | Metachronous | 1.64 | 1.26 – 2.14 | **0.0002** |

**Table 3_S. Univariate analyses for OS of previous anticancer treatments**

| **Characteristic** | | **Overall Survival** | | |
| --- | --- | --- | --- | --- |
|  |  | **HR** | **95% CI** | **p** |
| Previous anticancer therapy for non-metastatic disease: adjuvant | No | Ref. |  |  |
|  | Yes | 1.83 | 1.34 – 2.50 | **0.0002** |
| Previous anticancer therapy for non-metastatic disease: neo-adjuvant | No | Ref. |  |  |
|  | Yes | 2.26 | 1.39 – 3.68 | **0.0011** |
| Previous lines for metastatic  Disease* | 0 | Ref. |  |  |
|  | ≥1 | 2.06 | 0.85 – 4.99 | 0.1106 |
| Previous anticancer 1^ST^ line  therapy with Gemcitabine alone | No | Ref. |  |  |
|  | Yes | 1.15 | 0.57 – 2.32 | 0.7045 |
| Previous anticancer 1^ST^ line  therapy with Gemcitabine plus Nab -Paclitaxel | No | Ref. |  |  |
|  | Yes | 0.96 | 0.71 – 1.29 | 0.7836 |
| Previous anticancer 1^ST^ line  therapy with FOLFIRINOX | No | Ref. |  |  |
|  | Yes | 1.20 | 0.78 – 1.87 | 0.4086 |
| 1^ST^line: Disease  Control Rate (Recist v 1.1.) | No | Ref. |  |  |
|  | Yes | 0.83 | 0.64 – 1.09 | 0.1759 |
| Radiotherapy on primary T | No | Ref. |  |  |
|  | Yes | 0.78 | 0.56 – 1.10 | 0.1629 |
| Biliary stenting any time | No | Ref. |  |  |
|  | Yes | 1.22 | 0.93 – 1.58 | 0.1476 |

**Table 4_S. Univariate analyses for OS of baseline characteristics and major clinical features at start of treatment with Nal-IRI-5FULV**

| **Characteristic** | | **Overall Survival** | | |
| --- | --- | --- | --- | --- |
|  |  | **HR** | **95% CI** | **p** |
| Baseline ECOG PS | 0 | Ref. |  |  |
|  | ≥1 | 1.54 | 1.21 – 1.96 | **0.0004** |
| Baseline CA 19.9* | Normal | Ref. |  |  |
|  | > ULN (37 ng/mL) | 1.86 | 1.35 – 2.58 | **0.0002** |
| BMI | ≤ 18.5 | Ref. |  |  |
|  | > 18.5 | 0.87 | 0.65 – 1.18 | 0.3843 |
| Number of metastatic sites | 1 | Ref. |  |  |
|  | >1 | 1.33 | 1.04 – 1.70 | **0.0211** |
| Neutrophil-to-lymphocyte ratio (NLR)* | ≤ 5 | Ref. |  |  |
|  | > 5 | 2.39 | 1.75 – 3.27 | **0.0080** |
| Platelets | ≤150 | Ref. |  |  |
|  | >150 | 0.85 | 0.57 – 1.26 | 0.4108 |
| Albumin* | <4 g/dL | Ref. |  |  |
|  | ≥4 g/dL | 0.94 | 0.70 – 1.25 | 0.6664 |
| Total Bilirubin | ≤ 17 umol/L | Ref. |  |  |
|  | > 17 umol/L | 1.20 | 0.39 – 3.76 | 0.7500 |

**Table 5_S. Univariate analyses for OS of Nal-IRI-5FULV administration toxicity and efficacy**

| **Characteristic** | | **Overall Survival** | | |
| --- | --- | --- | --- | --- |
|  |  | **HR** | **95% CI** | **p** |
| Nal_IRI Line* | I | Ref. |  |  |
|  | II | 1.95 | 0.80 – 4.75 | 0.1393 |
|  | III | 2.39 | 0.96 – 5.97 | 0.0613 |
|  | IV | 2.85 | 0.90 – 9.00 | 0.0741 |

*The assumption of risks’ proportionality is not respected.
